# Reconstructing the Spatial Parameters of a Laser Beam Using the Transport-of-Intensity Equation

**DOI:** 10.3390/s22051765

**Published:** 2022-02-24

**Authors:** Michael Kovalev, Iliya Gritsenko, Nikita Stsepuro, Pavel Nosov, George Krasin, Sergey Kudryashov

**Affiliations:** 1 Laser and Optoelectronic Systems Department, Bauman Moscow State Technical University, 2nd Baumanskaya St. 5/1, 105005 Moscow, Russia; m.s.kovalev@bmstu.ru (M.K.); gritsenkoiv@student.bmstu.ru (I.G.); pan@bmstu.ru (P.N.); 2 Lebedev Physical Institute, Russian Academy of Sciences, Leninskiy Prospekt 53, 119991 Moscow, Russia; krasingk@lebedev.ru (G.K.); sikudr@lebedev.ru (S.K.)

**Keywords:** laser beam, wavefront, measurement spatial parameters, phase distortions, transport-of-intensity equation

## Abstract

A simple method for reconstructing the spatial parameters of a laser beam, based on the transport-of-intensity equation, is presented. Registration of cross-section intensity distributions in several planes was carried out using a single CMOS camera. The processing of the experimental measurements with the help of specialized software helped to reconstruct all of the spatial parameters, namely, the radius and position of the waist, Rayleigh length, angular divergence, quality parameter $M^{2}$ The method was compared with measurements made according to the international standard ISO 11146 and showed that the difference in the spatial parameters is 10% or less, which shows good agreement.

## 1. Introduction

When characterizing conditionally static wave fields of light beams, their complex amplitude is usually represented in the form of two related components—amplitude (intensity) and phase distributions. A reliable estimation of the phase component of the field, often referred to as wavefront reconstruction [1,2,3] or a measurement of its aberrations [4,5], is usually the most difficult procedure. This is because one has to face problems of a possible ambiguity of the phase reconstruction [6], the presence of singular regions [7], a search for optimal boundary conditions [8] and limitations on the admissible dynamic range of phase gradients [9], which is typical for a particular measuring instrument.

Meanwhile, the coherent radiation formed by stable configurations of laser resonators is well described by a special class of light beams—Gaussian beams. Such beams have a field amplitude distribution in the form of Hermite–or Laguerre–Gauss functions, with a phase surface that has a paraboloid of rotation. In laser engineering and technologies, the difference of the spatial parameters of a real laser beam from an ideal Gaussian beam is characterized by the beam quality parameter $M^{2}$.

Properties characterizing the propagation process of each laser beam are described by ten independent parameters [10,11]. The theoretical basis for finding these parameters is the method of determining second-order moments. However, due to the symmetry of most laser beams, which are widespread in many applications, their complete description requires fewer parameters, including the radius and position of the waist, Rayleigh length, angular divergence, quality parameter $M^{2}$. When measuring the spatial and energy characteristics and parameters of laser radiation, it is necessary to form a beam with a valid waist to measure intensity distributions in at least 10 sections, of which five measurements should be taken near the waist (left and right of the waist plane). Then, for each section, the normalized moments of intensity distribution $\sigma_{x}^{2}\left(z\right)$, $\sigma_{y}^{2}\left(z\right)$ and the effective beam radius $w_{eff}\left(z\right)=\sqrt{2\left(\sigma_{x}^{2}\left(z\right)+\sigma_{y}^{2}\left(z\right)\right)}$. By approximating the measurement results via a hyperbolic relationship, all the spatial characteristics of the laser beam above are calculated. However, despite the fact that the presented method is simple in its implementation, it is labor-intensive due to the need for multiple registrations of intensity distributions in the field under study.

Therefore, a number of alternative methods have been developed for more efficient $M^{2}$ measurements, including methods based on wavefront sensors [12,13], liquid crystal lens [14], computer-generated holograms [15], spatial light modulators [16], and several others.

At the same time, a method of real-time reconstruction of the complex amplitude exists for measuring the $M^{2}$ parameter by means of transport-of-intensity equation [17,18,19]. The transport-of-intensity equation (TIE) provides a non-interferometric way to obtain quantitative information on the phase of light beams by recording transverse field intensity distributions in two or more cross-sections [20,21]. The downside of the rapidity is that the measurement scheme requires two identical cameras [17], which will lead to additional errors and phase overruns, the magnitude of which will depend on the degree of identity of the selected cameras, the quality of the scheme assembly/alignment and the accuracy of the manufacture of the individual optical components. The disadvantages of the proposed approach in [18] include a large number of adjustable parameters, including the choice of the step between planes *dz*, the value of the shape factor for implementing the radial basis function (optimal approximation of the phase) and the Savitsky-Golay filter parameters. The determination of the optimal values of each of these parameters is a separate and very time-consuming task.

Therefore, this paper proposes a simple method for reconstructing the spatial parameters of a laser beam based on solving the TIE, using only one camera and a minimum number of optical elements. The method was compared with measurements made according to the international standard ISO 11146.

## 2. Methods and Approaches

**Figure 1:**
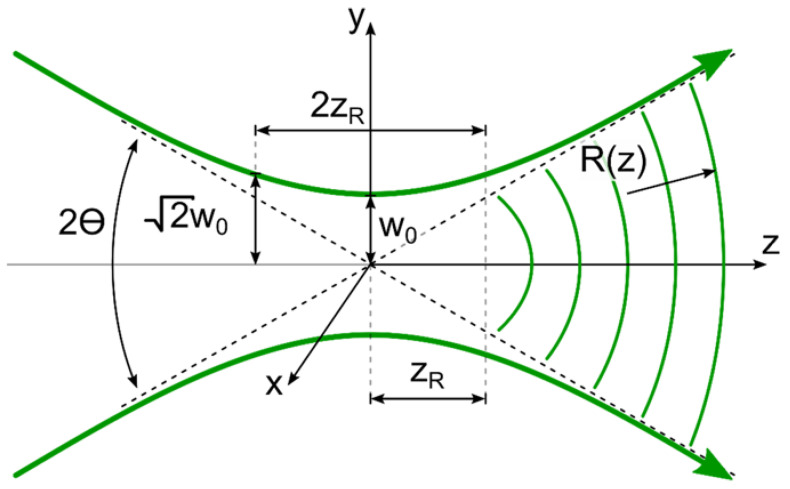
Spatial structure of the laser beam.

Figure 1 shows a longitudinal section of an axisymmetric laser beam propagating along the *z* axis, with a waist diameter $2w_{0}$ at a point with coordinate *z* = 0. The envelope (caustics) of the axisymmetric laser beam along the $1/e^{2}$ level of the total flux is a single-cavity hyperboloid of rotation, and the phase surface represents a paraboloid of rotation.

Since the processes of lase–matter interaction are three-dimensional, not only is the diameter of the focused spot (waist diameter) important, but also the characteristic waist length of the beam or, as experts in laser material processing term it, “depth of focus” or “waist length”. This value is determined by the Rayleigh length of the laser beam $z_{R}$ and is its doubled value (see Figure 1).

In turn, the Rayleigh length is the distance from the waist of the beam at which the beam size increases √2 times, i.e., the radiation power density at a distance $\pm z_{R}$ from the beam waist decreases by a factor of 2. Therefore, in the longitudinal direction, the laser beam can be divided into three parts. In the central part, for $\left|z\right|\leq z_{R}$, the transverse dimensions of the beam vary relatively little with changes in *z*. In the two peripheral parts (for $z<-z_{R}$ and $z>z_{R}$), however, the dimensions of the transverse beam increase noticeably with increasing z and are proportional to |z| for larger *z*. Therefore, the Rayleigh length of the laser beam also determines the so-called «near zone length» of the beam.

The dependences of the laser beam radius w(z) and the radius of curvature of its wavefront *R(z)* on the longitudinal coordinate *z* are described by the following equations [22]:

(1) $$w\left(z\right)=w_{0}\sqrt{1+{\left(\frac{z}{z_{R}}\right)}^{2}},\;$$

(2) $$R\left(z\right)=z+\frac{z_{R}^{2}}{z},$$

where *z* coordinate is measured from the beam waist position.

In order to characterize lasers whose beam quality is far from ideal (including high-power fiber and disk lasers), laser manufacturers widely use the Beam Parameter Product (BPP), [mm∙mrad]:

(3) $$BPP=w_{0}\theta =M^{2}\frac{\lambda }{\pi }.$$

In this relation, λ denotes the wavelength of the laser radiation; $M^{2}$ is a dimensionless parameter that determines the quality of a beam and is usually defined as the ratio of the BPP of a real laser beam to that of a diffraction-limited Gaussian beam at the same wavelength. For an ideal TEM_00_ Gaussian beam, $M^{2}=1$, and for a real beam $M^{2}>1$.

Rayleigh length and angular divergence of the beam are calculated using the following equations:

(4) $$z_{R}=\frac{\pi w_{0}^{2}}{M^{2}\lambda }=\frac{w_{0}^{2}}{BPP},\;$$

(5) $$2\theta =\frac{2w_{0}}{z_{R}}=2\frac{BPP}{w_{0}}.$$

These equations show that for a laser beam with a given wavelength λ and parameter $M^{2}$, one cannot simultaneously reduce the waist size and the angular divergence of the beam. Thus, decreasing the waist size results in decreasing the Rayleigh length and increasing the angular divergence. At the same time, it is possible to reduce only the waist size and angular divergence by increasing the refractive index of the medium in which the beam is formed. In addition, increasing the BPP of the beam increases the diameter of the focused spot and decreases the length of the divergence with respect to the ideal Gaussian beam, for which $BPP_{G}=\lambda /\pi$.

### 2.1. ISO Method

When processing the measurement results, it is convenient to represent the hyperbolic dependence of the laser beam radius as $w\left(z\right)=\sqrt{a+bz+cz^{2}}$ [10]. The coefficients *a*, *b*, *c* could be found by the least squares method using the following matrix expression [23]:

(6) $$\left(\begin{array}{c} a\\ b\\ c \end{array}\right)={\left(\begin{array}{ccc} n & \sum_{i=1}^{n}z_{i} & \sum_{i=1}^{n}z_{i}^{2}\\ \sum_{i=1}^{n}z_{i} & \sum_{i=1}^{n}z_{i}^{2} & \sum_{i=1}^{n}z_{i}^{3}\\ \sum_{i=1}^{n}z_{i}^{2} & \sum_{i=1}^{n}z_{i}^{3} & \sum_{i=1}^{n}z_{i}^{4} \end{array}\right)}^{-1}\cdot \left(\begin{array}{c} \sum_{i=1}^{n}w_{i}^{2}\\ \sum_{i=1}^{n}z_{i}w_{i}^{2}\\ \sum_{i=1}^{n}z_{i}^{2}w_{i}^{2} \end{array}\right)\;$$

where $w_{i}$ is a beam radius in the plane with coordinate $z_{i}$; *n* is a number of planes in which the radiation power density distribution was measured (n≥3); «−1» denotes inverse matrix notation. Using the coefficients *a*, *b*, *c*, we calculate the spatial parameters of the beam, the relationship of which is shown in Table 1.

### 2.2. TIE Method (Proposed Method)

In turn, the calculation of the curvature radius of the wavefront can be based on the solution of the TIE, in which the field phase function $\phi \left(r_{⊥},z\right)$ is related to the intensity distributions $I\left(r_{⊥},z\right)$ by the following equation [19,24]:

(7) $$\phi \left(r_{⊥},z\right)=-k\;\cdot \nabla^{-2}\left\{\nabla \cdot \left\{\frac{1}{I\left(r_{⊥},z\right)}\nabla \nabla^{-2}\frac{\partial I\left(r_{⊥},z\right)}{\partial z}\right\}\right\}.$$

One of the most popular methods for solving this equation is the method based on expressing differential operators through the Fourier transform [25], that is,

(8) $$\phi \left(r_{⊥},z\right)=-k\cdot {ℱ}^{-1}\left\{k_{x,y}\cdot ℱ\left\{\left[\frac{1}{I\left(r_{⊥},z\right)}\cdot {ℱ}^{-1}\left\{k_{x,y}\cdot ℱ\left\{\frac{\partial I\left(r_{⊥},z\right)}{\partial z}\right\}\right\}\right]\right\}\right\},\;\;k_{x,y}=\frac{jk_{x}+jk_{y}}{{\left(jk_{x}\right)}^{2}+{\left(jk_{y}\right)}^{2}},$$

where $r_{⊥}=\left(x,y\right)$ is the radius-vector; ℱ and ${ℱ}^{-1}$ are the direct and inverse two-dimensional Fourier transform, respectively, and $k_{x,y}=2\pi \nu_{x,y}$, where $\nu_{x,y}$ is the coordinate grid in the frequency domain; k=2π/λ is the wave number.

**Figure 2:**
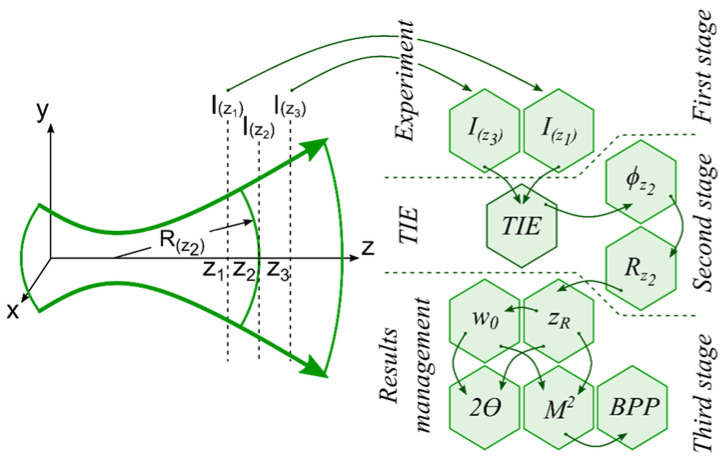
Schematic of the experimental measurements and algorithm for calculating the spatial parameters of the laser beam.

Figure 2 shows a scheme of experimental measurements and an algorithm for calculating the spatial parameters of the laser beam by solving the TIE, which consists of three main stages. At the first stage, two intensity distributions $I\left(r_{⊥},z_{1}\right)$ and $I\left(r_{⊥},z_{3}\right)$ are recorded using a camera. Then, at the second stage, the phase $\phi \left(r_{⊥},z\right)$ is calculated using the registered intensity distributions by solving the TIE, and then the wavefront curvature radius $R_{z_{2}}$ is calculated using the geometric method based on the phase data [19]. At the last stage, by the least-squares method the spatial parameters of the laser beam are calculated from the values of $R_{z_{2}}$ according to the relations given in Table 1.

## 3. Experimental Part

As a rule, for the experimental measurement of all spatial parameters of the beam, an additional optical system (lens) was used and radiation intensity distributions were measured in several cross sections of the beam transformed by this lens. Therefore, in the experiment, horizontally polarized radiation from a Satsuma femtosecond laser with a second harmonic wavelength $\lambda_{las}=515.6$ nm, pulse duration of 10 ps, and frequency of 10 kHz was focused, using a quartz flat-convex lens with a focal distance f′=550 mm. The specifications for the Satsuma femtosecond laser are shown in Table 2.

**Figure 3:**
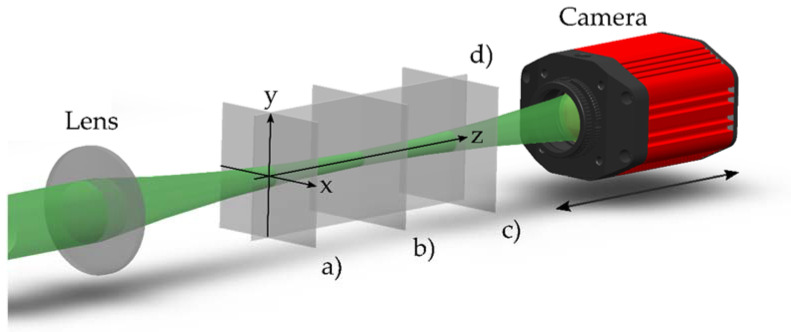
Experimental scheme consisting of a focusing lens and a CMOS-camera. The planes (**a**–**c**) show the cross-sections of the intensity distributions captured along the propagation axis. The plane (**d**) marks the longitudinal section of the intensity distribution.

**Figure 4:**
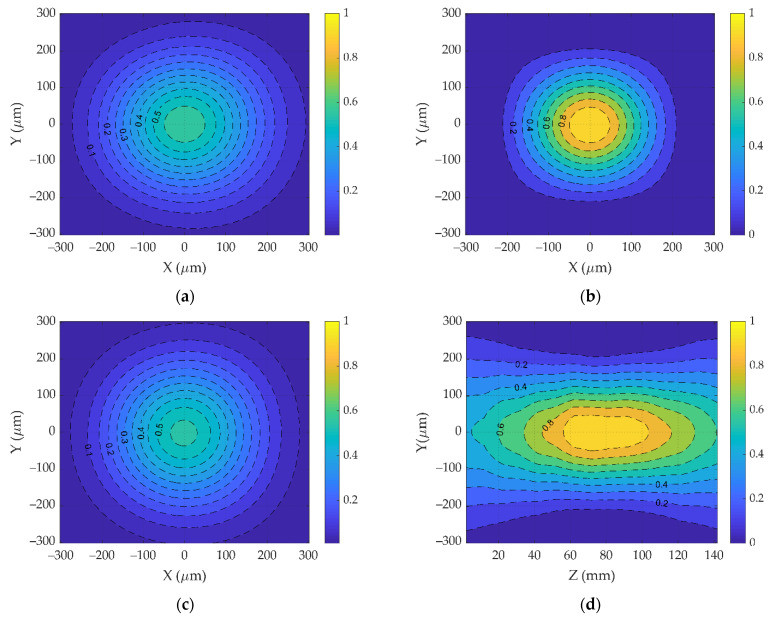
Cross-sections of the intensity distribution in planes with (**a**) Z = 22 mm; (**b**) Z = 82 mm; (**c**) Z = 140 mm; (**d**) Reconstructed longitudinal intensity distribution.

The intensity distributions were registered using a CMOS camera (1920 × 1080 pixels with dimensions 5.04 × 5.04 μm) mounted on a linear motion platform that moved the CMOS camera along the radiation propagation axis (Figure 3). A view of the two-dimensional sections of the intensity distribution in the X0Y (a), (b) and (c) planes obtained using the camera is shown in Figure 4a–c, respectively. Based on such intensity distributions, the longitudinal intensity section of the axisymmetric laser beam was reconstructed (Figure 4d).

## 4. Results and Discussion

### 4.1. ISO Method

As noted above, the envelope (caustic) of an axisymmetric laser beam in terms of the level $1/e^{2}$ of the total flux is a single-cavity hyperboloid. Based on the obtained intensity distribution in the Y0Z plane (Figure 4d), one can approximate and represent the dependence for the laser beam radius (axis in Y) as $w\left(z\right)=\sqrt{a+bz+cz^{2}}$ [10]. A total of 143 cross sections obtained in the experiment with a step of 1 mm were used to construct the envelope. The coefficients *a*, *b*, *c* were found using the matrix expression (4) and were equal to the following: a=0.058 mm^2^, $b=-1.307\times 10^{-3}$ mm, $c=7.866\times 10^{-6}$ for plane X0Z and a=0.059 mm^2^, $b=-1.458\times 10^{-3}$ mm, $c=9.506\times 10^{-6}$ for plane Y0Z. Then, these coefficients were used to calculate the spatial parameters of the beam, the relationship of which is given in Table 1. The numerical results are presented in Table 3.

### 4.2. TIE Method (Proposed Method)

To find the spatial parameters of the laser beam by the proposed method, it is first necessary to register at least three transverse intensity distributions in arbitrary planes. In this case, the extreme planes will be denoted as $z_{1}$ and $z_{3}$, and the intermediate one between them is $z_{2}$. For a comparative analysis of the methods, we used the same experimental data obtained for the ISO method.

**Figure 5:**
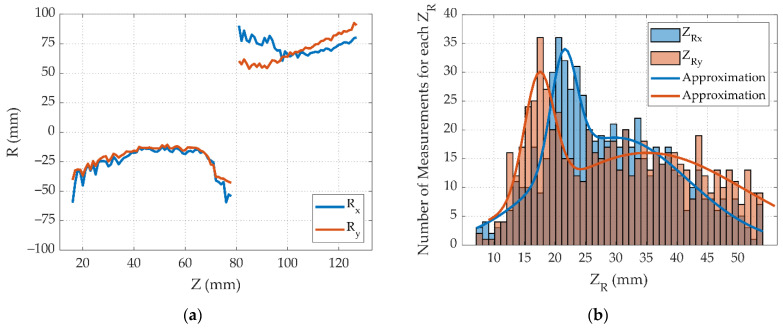

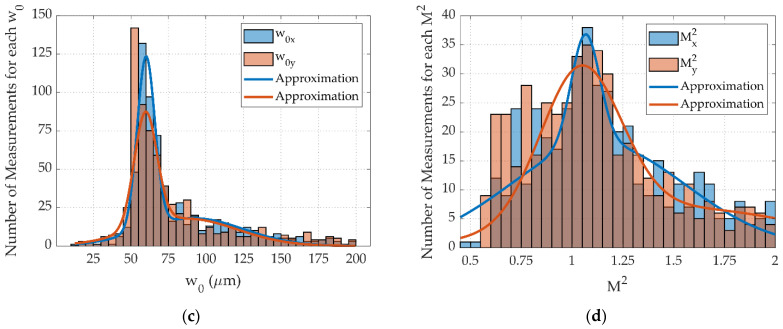
Numerical calculations of (**a**) Radius of curvature $R\left(z_{2}\right)$ depending on the longitudinal coordinate; (**b**) Rayleigh length $z_{R}$; (**c**) Waist radius $w_{0}$; (**d**) Parameter $M^{2}$.

At the second stage, the phase function of the field $\phi \left(r_{⊥},z_{2}\right)$ was calculated using TIE for the intensity distributions in the two extreme planes $z_{1}$ and $z_{3}$ (the distance between the planes $\Delta z_{1-3}$ was chosen arbitrarily and amounted to 30 mm). The resulting phase function of the field $\phi \left(r_{⊥},z_{2}\right)$ in the intermediate plane $z_{2}$ was used for the approximation and calculation of the wavefront curvature radius $R\left(z_{2}\right)$ [19]. The radius of curvature was calculated for the different pairs of planes $z_{1}$ and $z_{3}$ from the obtained dataset. The results are shown in Figure 5a.

Next, the values of the Rayleigh length $z_{R}$ were obtained for the longitudinal planes X0Z and Y0Z using Equation (2). Figure 5b shows a histogram of the obtained values with lines showing their approximation. Then, the waist radius $w_{0}$ was calculated using Equation (1). The results of the calculation are presented in Figure 5c. After that, the parameter $M^{2}$ was calculated according to Equation (4). The results are shown in Figure 5d. The comparison of the spatial parameters reconstructed by the two methods is shown in Table 3. It can be seen that the methods are in good agreement with each other.

It should be noted that in this work we investigated the performance of our method with a single-mode single-frequency laser, with parameter $M^{2}$ close to 1. We will continue our research, and in the near future we plan to study the multimode beams. The purpose of this research will be to analyze the boundaries of the method applicability. We aim to configure where, first of all, it will be necessary to measure the stability of the method to fluctuations of the output laser power. However, we can already argue that the proposed method is not inferior to the ISO 11146 one in terms of measuring $M^{2}$ close to 1 (Table 3), while being easier to implement. Additionally, the proposed method is not inferior to existing commercial solutions, for example, the Thorlabs M2MS system.

The measurement time of parameter $M^{2}$ amounted to 30 s, which is optimal, yet with current algorithmic solutions it can be further optimized to achieve better performance. The expected improvement from the algorithm optimization can reduce the measurement time considerably. Additionally, in combination with the simple installation of the elements required for the implementation of the method, the method can become a widely used alternative solution for many photonics problems.

## 5. Conclusions

This work presents a simple method for reconstructing all of the laser beam spatial parameters based on the solution of the TIE. The phase function of the field can be quickly recovered by solving the TIE and does not require the use of a complex optical system. The method was compared with measurements made according to the international standard ISO 11146 and showed that the difference of the spatial parameters is 10% or less, which means that the methods are in good agreement with each other. The advantages of this approach are its experimental simplicity and the possibility to perform quick measurements without time-consuming numerical analyses. In addition, this method is suitable for determining the beam quality of a dynamic laser system.

## Figures and Tables

**Table 1:** Functional dependences of spatial parameters of the laser beam on *a*, *b*, *c*.

Parameters	Equations
Radius of the laser beam waist	$w_{0}=\frac{\sqrt{4ac-b^{2}}}{2\sqrt{c}}$
Position of the beam waist relative to the selected reference plane (taking into account the sign rule adopted in optics)	$S_{0}=-\frac{b}{2c}$
Rayleigh length	$z_{R}=\frac{\sqrt{4ac-b^{2}}}{2c}$
Angular divergence of the beam	$2\theta =2\sqrt{c}$
BPP	$BPP=\frac{\sqrt{4ac-b^{2}}}{2}$

**Table 2:** Datasheet for the Satsuma laser.

Parameters	X0Z	Y0Z	Comment
Central wavelength, (nm)	1031.2		
Beam diameter, (mm)	1.627	1.576	at 60 mm from exit
Beam ellipticity, (%)	3		at 60 mm from exit
Quality parameter $M^{2}$	1.075	1.042	
Astigmatism (%)	0.2		
Beam divergence, (mrad)	1.424	1.383	full angle

**Table 3:** Spatial parameters of the Satsuma laser beam.

Parameters	TIE (X0Z)	TIE (Y0Z)	ISO 11146 (X0Z)	ISO 11146 (Y0Z)
Radius of the laser beam waist (mm)	0.064	0.059	0.061	0.053
Beam waist position (mm)	83.087	77.014	83.306	76.686
Rayleigh length (mm)	21.606	17.576	21.785	17.251
Quality parameter $M^{2}$	1.066	1.051	1.061	1.047
Angular divergence of the beam (mrad)	0.55	0.58	0.482	0.485

## Data Availability

Data underlying the results presented in this paper are available from the corresponding author upon reasonable request.
